# 234. Safety and Immunogenicity of mRNA-1345, an mRNA-Based RSV Vaccine in Younger and Older Adult Cohorts: Results from a Phase 1, Randomized Clinical Trial

**DOI:** 10.1093/ofid/ofac492.312

**Published:** 2022-12-15

**Authors:** Grace L Chen, Runa Mithani, Archana Kapoor, Sophia Lu, Laila El Asmar, Catherine A Panozzo, Christine A Shaw, Sonia K Stoszek, Allison August

**Affiliations:** Moderna, Inc., Cambridge, Massachusetts; Moderna, Inc., Cambridge, Massachusetts; Moderna, Inc., Cambridge, Massachusetts; Moderna, Inc., Cambridge, Massachusetts; Moderna, Inc., Cambridge, Massachusetts; Moderna, Inc., Cambridge, Massachusetts; Moderna, Inc., Cambridge, Massachusetts; Moderna, Inc., Cambridge, Massachusetts; Moderna, Inc., Cambridge, Massachusetts

## Abstract

**Background:**

Respiratory syncytial virus (RSV) is a public health burden; no vaccine is currently available. An mRNA-based RSV vaccine (mRNA-1345) encoding the RSV prefusion stabilized F (preF) glycoprotein is under clinical investigation.

**Methods:**

A phase 1, randomized, observer-blind, placebo-controlled, dose-ranging study assessed safety and immunogenicity of mRNA-1345 in younger adults (YA; 18-49 years) and older adults (OA; 65-79 years) (NCT04528719). YA and OA were randomized to receive 1 dose of mRNA-1345 (50, 100, or 200 µg) or placebo.

**Results:**

In all, 74 YA participants (mRNA-1345, n=19-20; placebo, n=15) and 202 OA participants (mRNA-1345, n=47-48; placebo, n=59) received study injections. mRNA-1345 was well-tolerated in both groups, with lower reactogenicity observed in OA vs YA at higher doses. Injection site pain was the most frequent local solicited adverse reaction (SAR, YA: mRNA-1345, 73.7-100%; placebo, 0%; OA: mRNA-1345, 61.7-78.7%; placebo, 12.7% [Fig 1]). Erythema and swelling were less frequent (mRNA-1345: YA, 5.3-15.0%; OA, 0-4.3%; and YA, 5.0-15.0%; OA, 2.1-4.3%; respectively vs placebo 0% for all). Overall, 57.9-100% (YA) and 53.2-78.7% (OA) of mRNA-1345 and 40.0% (YA) and 45.5% (OA) of placebo groups reported ≥ 1 systemic SAR, most commonly headache, fatigue, myalgia, and arthralgia. As expected, neutralizing antibodies (nAbs) were present at baseline (BL; Fig 2); mRNA-1345 significantly boosted antibody titers through month (M) 1 in YA and OA, with comparable immunogenicity observed across age groups. M1 geometric mean fold rise (GMFR) for RSV-A nAbs were 20.0-22.3 (YA) and 12.1-16.6 (OA) and for RSV-B, nAbs were 11.7-14.4 (YA) and 8.7-12.6 (OA). M1 PreF binding antibody (bAb) GMFRs were 16.1-21.7 (YA) and 8.4-12.1 (OA; Fig 3). Peak antibody titers declined through M6, but levels remained ≥ 4.1-fold above BL with minimal dose response. M6 GMFR for RSV-A nAbs were 7.0-9.6 (YA) and 4.1-5.8 (OA) and for RSV-B, nAbs were 5.0-8.9 (YA) and 4.5-5.5 (OA). M6 PreF bAbs GMFR were 5.9-7.0 (YA) and 4.1-4.7 (OA). Antibody decline over time was comparable in YA and OA cohorts.

**Figure:**
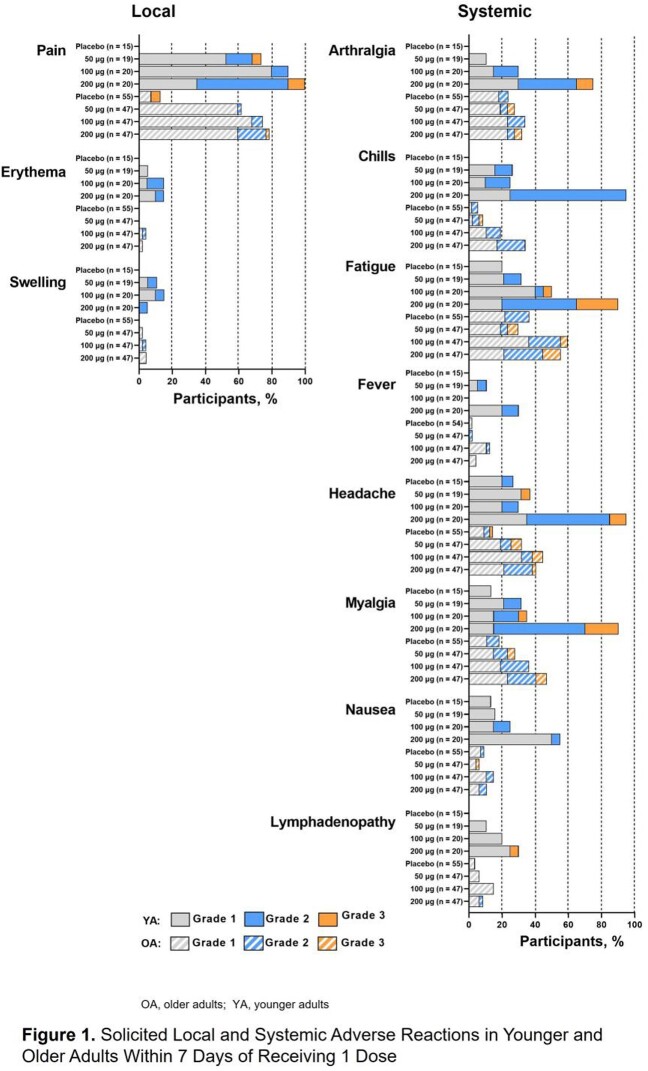

**Figure:**
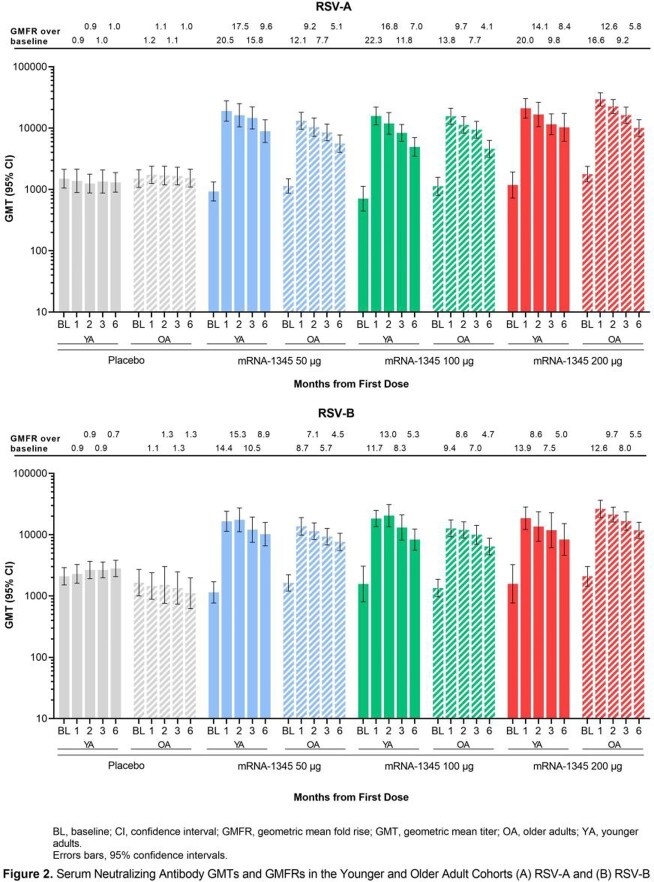

**Figure:**
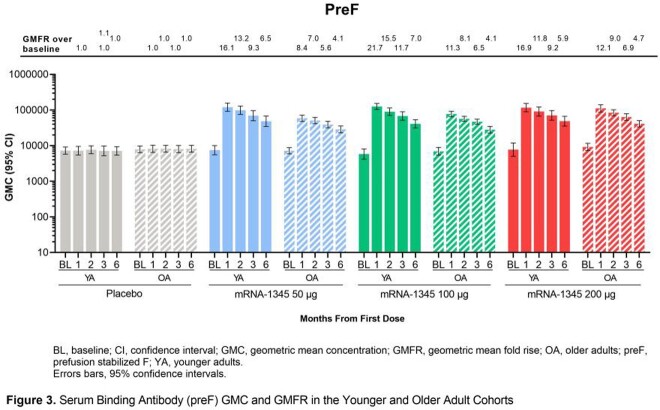

**Conclusion:**

mRNA-1345 is well-tolerated in YA and OA. Antibody levels were boosted substantially above BL through M6 in both cohorts. These data support the continued development of mRNA-1345 as an RSV vaccine.

**Disclosures:**

**Grace L. Chen, MD, MPH**, Moderna, Inc.: Salary|Moderna, Inc.: Stocks/Bonds **Runa Mithani, PharmD**, Moderna, Inc.: Salary|Moderna, Inc.: Stocks/Bonds **Archana Kapoor, PhD**, Moderna, Inc.: Salary|Moderna, Inc.: Stocks/Bonds **Sophia Lu, PhD**, Moderna, Inc.: Salary|Moderna, Inc.: Stocks/Bonds **Laila El Asmar, PhD**, Moderna, Inc.: Salary|Moderna, Inc.: Stocks/Bonds **Catherine A. Panozzo, PhD**, Moderna, Inc.: Salary|Moderna, Inc.: Stocks/Bonds **Christine A. Shaw, PhD**, Moderna, Inc.: Salary|Moderna, Inc.: Stocks/Bonds **Sonia K. Stoszek, PhD**, Moderna, Inc.: Salary|Moderna, Inc.: Stocks/Bonds **Allison August, MD**, Moderna, Inc.: Salary|Moderna, Inc.: Stocks/Bonds.

